# Clinical Features of Miller-Fisher Syndrome in Pregnancy

**DOI:** 10.1155/2015/840680

**Published:** 2015-12-01

**Authors:** Masanori Ono, Hideki Sato, Mayu Shirahashi, Noriko Tomioka, Julia Maeda, Keiko Watanabe, Tomoko Amagata, Toshiyuki Ikeda, Kazumi Yakubo, Tatsuro Fukuiya

**Affiliations:** ^1^Department of Obstetrics and Gynecology, Saitama City Hospital, Midori, Saitama 336-8522, Japan; ^2^Department of Neurology, Saitama City Hospital, Midori, Saitama 336-8522, Japan

## Abstract

Miller-Fisher syndrome (MFS) is recognized as a variant of Guillain-Barré syndrome (GBS). MFS is a rare disorder that is characterized by the acute onset of ophthalmoplegia, ataxia, and areflexia/hyporeflexia. MFS has a higher incidence in Asia, where the incidence is estimated to be 18%–26% of GBS compared with 3%–5% in the West. The differential diagnosis of MFS includes Wernicke's encephalopathy (WE) which is characterized by a clinical triad (nystagmus and ophthalmoplegia, mental status changes, and ataxia), myasthenia gravis, and brainstem stroke. The association between MFS and pregnancy has not been reported previously. Here, we describe the clinical features of a pregnant woman in early pregnancy with MFS. This case highlights the fact that it is necessary to establish an accurate diagnosis based on the details from the patient's history on appropriate complementary testing in a pregnant patient with MFS.

## 1. Introduction

Miller-Fisher syndrome (MFS) came to be recognized as a variant of Guillain-Barré syndrome (GBS) nearly 60 years ago [1]. MFS is a rare disorder that is characterized by the acute onset of ophthalmoplegia, ataxia, and areflexia/hyporeflexia [2]. The worldwide incidence of GBS is estimated at 1-2/100,000, and MFS represents a small fraction of that total. MFS has a higher incidence in Asia, where the incidence is estimated to be 18%–26% of GBS compared with 3%–5% in the West [1, 3–5]. The differential diagnosis of MFS includes Wernicke's encephalopathy (WE) which is characterized by a clinical triad (nystagmus and ophthalmoplegia, mental status changes, and ataxia), myasthenia gravis, and brainstem stroke [6]. WE can complicate hyperemesis gravidarum because of the hypermetabolic state of pregnancy, increased fetal demand, and poor intake due to nausea and vomiting [7]. The association between MFS and pregnancy has not been reported previously. The present report describes the clinical features of a pregnant woman in early pregnancy with MFS, in whom WE was ruled out.

## 2. Case Report

A 26-year-old woman at 11 weeks of gestation presented to the emergency department for evaluation of severe nausea, dizziness, and double vision. The physician who evaluated her in the emergency room referred her to our department with a tentative diagnosis of WE based on the early gestation, nausea, and dizziness. She had hyperemesis gravidarum since 6 weeks of gestation. She first noted double vision 4 days prior to the emergency room visit. On questioning, she admitted that the gait difficulties secondary to dizziness worsened slightly each day. Her double vision was now continuous, and objects appeared skewed. She reported that she was nauseated and found it difficult to tolerate the double vision. She reported no definite weakness but had considerable difficulty walking without assistance. She reported no paresthesias or sensory loss in her limbs, trunk, or face. She had an unremarkable medical history and she had not been hospitalized. Her family history was significant for maternal IgA nephropathy. The obstetric examination at the time of admission revealed an 11-week gestation and a single live fetus was detected* in utero*. On the general physical examination, she appeared uncomfortable, but not acutely ill. She tended to keep her eyes closed. She did not have a history of an upper respiratory or gastrointestinal tract infection. She was fully alert with a normal mental status examination. Her eyes had dysconjugate gaze, with markedly restricted abduction of the left eye, and modest restriction of movement of both eyes in all other directions. There was no nystagmus. There was no ptosis, and the pupils were equal and normally reactive. The degree of ocular misalignment did not appear to fluctuate. Facial strength and sensation were normal. The palate elevated normally, and the tongue appeared normal as well. Hearing was intact. The tone, bulk, and strength of her extremities were normal. Rapid alternating movements of the hands and feet were slow. She was imprecise when performing the knee-heel-shin maneuver. Vibratory and temperature perception were normal in her hands and feet, but proprioception was impaired. She could not walk or stand without assistance, despite appearing to have sufficient motor strength. Brain magnetic resonance imaging (MRI) revealed no areas of restricted diffusion or other signal abnormalities. A lumbar puncture demonstrated no remarkable findings. A needle electromyogram (EMG) of the left upper and lower extremity muscles was normal. Brainstem stroke was excluded by MRI. Myasthenia gravis was denied by lacking diurnal fluctuation of symptoms and easy fatigability.A diagnosis of MFS was suspected, and the patient was treated with supportive care. Several serologic studies were obtained, including a normal thyroid-stimulating hormone (TSH) level, a normal antinuclear antibody (ANA) titer, and a negative rapid plasma reagin (RPR). The anti-GQ1b antibody titer was markedly elevated. Additionally, the anti-GD1b and anti-GT1a antibody titers were elevated in this patient. Before establishing the diagnosis of MFS, thiamine was administered, but the response to thiamine was poor. She had ophthalmoplegia, ataxia, areflexia, and absence of limb weakness and hypersomnolence. After the diagnosis of MFS was established, we considered intravenous immunoglobulin (IVIG) or no treatment. Because the natural history of MFS is favorable, we recommended no treatment. At a follow-up visit approximately 6 weeks after symptom onset, she reported diplopia on left gaze and no gait unsteadiness. Her clinical course was monophasic with improvements not further than 8 weeks. Her antepartum course was otherwise unremarkable, and she delivered a healthy male at term, weighing 3520 g. The infant Apgar scores were 8 at 1 minute and 8 at 5 minutes. The umbilical arterial blood pH was 7.398. Seven months following symptom onset, she had returned to her baseline state of good health and was doing well. The deep tendon reflexes had returned to normal.

## 3. Discussion

Classic MFS is defined by the acute onset of ophthalmoparesis, ataxia, and areflexia/hyporeflexia [1, 2]. The features of MFS may also be present with signs and symptoms indicative of more widespread neuropathy. Antibodies directed against the GQ1b ganglioside are often present in patients with classic MFS [8–10]. Ophthalmoparesis is an early finding, often leading to diplopia, which is a frequent presenting symptom in patients with MFS. The presence of ophthalmoparesis in MFS is highly associated with the presence of antibodies to GQ1b ganglioside. Nishimoto et al. reported that antibody testing to GQ1b ganglioside was superior to a cerebrospinal fluid examination in supporting a diagnosis of MFS during the first 3 weeks of illness [9]. Pupillary abnormalities, indicative of internal ophthalmoparesis, are common in MFS. Findings may include pupillary asymmetry and sluggish reactivity to light [11].

The ataxia in MFS is often very severe. Patients are unable to ambulate independently, despite normal strength. Ataxia is occasionally seen in isolation and, similar to isolated ophthalmoparesis, is associated with antibodies to GQ1b ganglioside [12]. There is evidence of both central and peripheral mechanisms of ataxia in MFS. Proprioception is severely impaired, and muscle spindle afferent fibers appear to be particularly involved [13]. MFS is not typically associated with any abnormalities on brain imaging. Many patients with MFS do undergo brain imaging, particularly if the triad of symptoms is not fully present, and the vast majority have normal brain MRI studies [14]. In contrast, MRI studies in our patient are thought to be critical in making a differential diagnosis for WE. Typical MRI of WE usually shows symmetric T2 signal intensity alterations in the medial thalami, mammillary bodies, tectal plate, and the periaqueductal area [15].

Electrodiagnostic studies (nerve conduction studies and EMG), which play a large role in confirming the diagnosis of AIDP and other forms of GBS, have a more limited role in MFS. In patients with MFS, routine studies may be normal. Mild abnormalities in sensory nerve conduction studies often occur. Electrodiagnostic studies in our patient revealed normal findings.

There is growing recognition that antibodies directed against gangliosides play a significant role in the pathogenesis of many acute autoimmune neuropathies. This is particularly true of MFS. Approximately 80%–90% of MFS patients have antibodies against GQ1b [16]. Our patient had highly elevated anti-GQ1b antibodies in serum; this result distinguished MFS and WE in our patient with an early pregnancy.

This case highlights the fact that it is necessary to establish an accurate diagnosis based on the details from the patient's history on appropriate complementary testing in a pregnant patient with MFS.
